# Policy effectiveness evaluation of rural environmental sanitation governance in China: based on the PMC index model

**DOI:** 10.3389/fpubh.2025.1575719

**Published:** 2025-06-30

**Authors:** Yingda Xu

**Affiliations:** School of Law, Huazhong University of Science and Technology, Wuhan, Hubei, China

**Keywords:** policy effect evaluation, rural environmental sanitation, PMC index model, Policy effect, rural environmental policy

## Abstract

**Background:**

Rural environmental sanitation governance is of vital importance for improving rural living standards and narrowing urban-rural gaps in China. However, the effectiveness of existing policies remains suboptimal due to structural design flaws, highlighting the need for systematic evaluation to address these issues.

**Methods:**

This study integrates the “Policy Modeling Consistency (PMC-Index)” with text-mining technology to quantitatively assess 24 local policies from 7 Chinese cities, with 7 representative samples selected via stratified sampling. The evaluation framework comprises 9 primary variables and 37 subvariables, systematically analyzing the integrity of policy texts through a structured approach.

**Results:**

The findings reveal an average PMC index of 6.03 across the policies. Specifically, 1 policy is rated “excellent”, 4 “good”, and 2 “unsatisfactory”. Key deficiencies are identified in X_1_ (Policy Nature), X_2_ (Policy Timeliness), X_5_ (Policy Targets), and X_7_ (Policy Functions), indicating challenges such as insufficient feedback mechanisms, lack of mid-term planning, and limited stakeholder inclusivity.

**Conclusion:**

This study conducts a quantitative evaluation of rural environmental sanitation governance policies in 7 Chinese cities. Although the overall content design of these policies is basically reasonable, obvious deficiencies exist. To enhance policy effectiveness, it is necessary to construct a holistic framework integrating dynamic adjustment mechanisms and multi-stakeholder collaboration.

## 1 Introduction

Public policy evaluation is of utmost significance in assessing the rationality of national public actions (1). It refers to the process in which policy evaluation subjects, according to specific criteria and procedures, analyze and appraise the policy content through appropriate evaluation instruments, thereby establishing the groundwork for policy adjustment. Rural environmental governance is an indispensable task for enhancing the living standards of rural inhabitants and a fundamental approach to propelling the modernization of rural infrastructure construction and diminishing the urban–rural development gap (2). Given the restricted overall supply of infrastructure and basic public services in China, the public resources allocated to rural areas are preferentially dedicated to road facilities, irrigation installations, and drinking water facilities related to production activities. Meanwhile, providing living infrastructure and services, such as sanitary toilets, waste disposal stations, and sewage treatment equipment, essential for a rural living environment, is relatively inadequate (3). Local governments in China have, respectively, initiated rural environmental sanitation governance and introduced relevant policies to optimize the rural living milieu.

Nevertheless, considering the policy implementation outcomes, the overall efficacy of the existing rural environmental governance is underwhelming, and the effective utilization rate of funds is relatively low (4). Consequently, to further boost the effectiveness of policy support, it is imperative to systematically evaluate the rural environmental sanitation governance policies in diverse regions of China. In fact, due to the significant gap between policy objectives and actual achievements, an increasing number of scholars have studied China’s rural environmental sanitation governance policies. However, most extant studies center on the qualitative assessment of the overall policy content, overlooking the quantitative evaluation of the policy’s micro-components. Moreover, nearly all related research evaluates policies solely from the effectiveness perspective post-policy implementation, neglecting the consistency, merits, and demerits of the policy texts themselves. Therefore, to address the issues above, this study quantitatively evaluates China’s rural environmental sanitation governance policy texts from a micro perspective to indicate the direction for policy adjustment.

The principal contributions of this research are as follows. Firstly, by harnessing text-mining technology, it conducts an in-depth exploration of the rural environmental sanitation governance policies in major cities in China and performs a refined and systematic evaluation of the policies’ specific content. Secondly, based on the PMC index model, it devises a quantitative evaluation framework for China’s rural environmental sanitation governance policies, thereby contributing a novel method to existing research. Thirdly, it selects seven policies as research samples, analyzes the pros and cons of each policy, and furnishes specific solutions for the adjustment of China’s rural environmental sanitation governance policies.

## 2 Literature review

Research on China’s rural environmental sanitation governance policies can be classified into two categories. However, to gain a more comprehensive understanding of the field, it is also crucial to consider the latest international research trends and the background of the indicator systems used in evaluations.

### 2.1 Policy content and evolution

Some research evaluates policies through close examination of their content and evolutionary trajectories. Predominantly qualitative in methodology, this approach exhibits a notable scarcity of quantitative studies. In the international arena, recent studies have also explored policy content and evolution in rural environmental sanitation. For example, Smith et al. (5) used a mixed-methods approach to analyze rural environmental policies, highlighting the importance of policy flexibility in adapting to local contexts. Khatibiet et al. (6) conducted a longitudinal study on rural environmental policy changes, identifying key drivers such as public awareness and technological advancements. These international studies provide a broader perspective that can be compared and contrasted with Chinese research.

Hu et al. (7) comprehensively assessed rural environmental sanitation governance policies in China. Applying historical institutionalism and policy dynamics lenses, they investigated the underlying significance and drivers of policy evolution while projecting future developmental trends. He et al. (8) based their study on the “fuzzy-conflict” analysis framework and applied the comparative analysis approach. Subsequently, they conducted in-depth investigations into the content of China’s rural environmental sanitation governance policy texts. They proposed that, due to the ambiguity inherent in policy texts and the conflicts with local government functions, rural ecological policies will likely fall into “institutional idling” during the implementation stage. Lan et al. (9) constructed a theoretical framework integrating three key factors: organization, system, and value. Using a causal-process-tracing method based on theoretical construction, they analyzed typical cases of rural environmental sanitation governance policies in China. They proposed the establishment of a stable ecological policy implementation network for rural areas.

Collectively, these studies provide foundations for policy content indicator systems. However, developing such systems must also consider their methodological evolution. Initially reliant on fundamental qualitative indicators, using policy objectives and phrasing, rural environmental sanitation evaluation frameworks have progressively incorporated comprehensive quantitative metrics under demands for precise policy assessment. Contemporary systems integrate multidimensional policy attributes, including clarity, comprehensiveness, and alignment with national/global environmental objectives, by synthesizing cross-regional and interdisciplinary best practices to ensure efficacy and applicability.

### 2.2 Policy implementation effectiveness

In contrast, the second major approach is to explore and assess the’ effectiveness of policies from the policy implementation perspective. This kind of research mainly adopts quantitative empirical research methods. Research on policy implementation effectiveness in rural environmental sanitation has also been active internationally. Secchi et al. (10) evaluated the implementation of rural environmental policies using a cost–benefit analysis approach, emphasizing the importance of stakeholder participation for successful implementation. Horn et al. (11) used a social-network analysis method to study the implementation process of rural environmental policies, revealing the role of local community networks in policy diffusion. These international studies offer valuable insights that can enrich the understanding of policy implementation effectiveness in China.

Tang et al. (12) analyzed the logical connection between environmental policies and farmers’ behaviors from a game theory perspective. The research findings indicated that environmental policies can stimulate farmers to participate in rural environmental governance. However, the positive influence of these policies gradually emerges only when farmers’ household income reaches a certain threshold. Yan et al. (13) used the knowledge-network analysis tool CiteSpace to thoroughly analyze China’s rural environmental governance policies’ fundamental theories, research hotspots, and evolutionary trends. They took 2,783 pieces of literature from 1998 to 2020 as samples. Although their research mainly focused on literature analysis and may have overlooked some practical implementation issues in rural areas, it still revealed that China’s environmental governance research has made remarkable progress. Teng et al. (14) categorized ecological environment policies into production environment policies and living environment policies. By combining regression analysis with fuzzy set qualitative comparative analysis, they explored the impact of the linkage and matching relationships of ecological environment policies on the conscious pro-environmental behaviors of rural residents from the perspective of the interaction between production and living environment policies.

In summary, studies using the second approach to evaluate policy implementation effectiveness aim to measure policies’ real-world outcomes and impacts in practical scenarios. Such research involves multiple factors, including environmental quality changes, changes in farmers’ behaviors, and economic effects. Additionally, by optimizing research designs based on real-world experiences and feedback from policy implementers and stakeholders, these studies can provide a foundation for constructing a policy evaluation indicator system.

### 2.3 Overall review and the significance of the PMC index model

Overall, the existing research has made substantial contributions. It has contributed to improving China’s rural environmental sanitation governance policies.

However, it is crucial to critically discuss the limitations of existing qualitative evaluations and post implementation evaluations. In qualitative evaluations, the analysis often relies on subjective interpretations and expert judgments, which may lead to inconsistent evaluation results due to differences in researchers’ perspectives and experiences. Moreover, qualitative studies usually focus on specific cases or regions, lacking the ability to generalize findings to a broader context. As for post implementation evaluations, they often face challenges in accurately measuring the long term impacts of policies, as many influencing factors are difficult to isolate and quantify. Additionally, post implementation evaluations may not comprehensively consider the complex interactions between different policy elements, resulting in an incomplete understanding of policy effectiveness. From the quantitative research perspective, the current focus of relevant policies’ quantitative evaluation lies in the study of policy effectiveness, while overlooking the quantitative analysis of the completeness and consistency of the policy content itself (15).

Currently, mainstream policy text analysis methods primarily include content analysis, discourse analysis, and frame analysis. Content analysis quantifies textual features through systematic coding and statistical approaches; discourse analysis focuses on power relations and ideology underlying texts; frame analysis emphasizes the excavation of cognitive frameworks constructed within texts. In comparison, the PMC Index Model demonstrates its advancements in two key aspects: The evaluation framework developed by the PMC Index Model is broader in coverage and more comprehensive, as it not only focuses on textual content but also incorporates considerations of textual frameworks. On the other hand, it presents research outcomes through three-dimensional (3D) charts, offering a more intuitive and concrete representation compared to the two-dimensional (2D) charts used in other research methods to display results.

Compared with these methods, the PMC index model evaluation method has more conspicuous advantages (16), mainly for the following reasons:

First, to address the subjectivity issue in qualitative evaluations, the evaluation indicators of the PMC index model are derived from policy-text mining, which effectively avoids the subjectivity of manual extraction and induction, and thus has strong objectivity and accuracy.

Second, unlike qualitative studies with limited generalization ability, the PMC index model does not deliberately differentiate the importance of various levels of indicators and does not restrict the number and weight of variables, which is conducive to identifying all potential influencing factors and enables more generalizable results.

Third, in contrast to the challenges in measuring long term impacts in post-implementation evaluations, the PMC index model can systematically analyze policy content, helping predict potential policy effects and evaluate policies more comprehensively.

Fourth, compared to the possible lack of consideration of policy element interactions in post-implementation evaluations, presenting the numerical values of various policy indicators in a three-dimensional graph by the PMC index model enables more intuitive observation and comparison of the advantages and disadvantages of different policies, thus facilitating a more comprehensive understanding of policy effectiveness. The PMC index model also features higher analysis efficiency and stronger operability and practicality.

Recent research shows that the PMC index model has been extensively applied in evaluating public policy texts in fields such as the new energy vehicle industry, railway green construction, and public health event response. The abovementioned circumstances demonstrate that applying the PMC index model to evaluate China’s rural environmental sanitation governance policies is feasible, necessary, and highly scientific. Therefore, this study endeavors to expand the application scope of the PMC index model and utilize it to evaluate the completeness and consistency of China’s rural environmental sanitation governance policies.

## 3 Samples and methods

### 3.1 Overview of China’s rural environmental sanitation governance policies

The rural environmental sanitation governance policy is crucial in driving the advancement of rural living environment improvement initiatives in China (17). Ever since the start of the reform and opening-up policy, under the guidance of the rural environmental sanitation governance policy, the rural landscape in China has witnessed dramatic transformations. The living conditions, average life expectancy, and the overall quality of the rural population have also been substantially improved.

In recent years, the Chinese government has successfully rolled out a series of supportive policies to strengthen the governance of the rural living environment and meet the growing demands of rural residents for a healthier environment. In 2010, the No. 1 Central Document proposed the need to “effectively manage garbage and sewage treatment and upgrade the rural living environment” (18). In 2014, the General Office of the State Council issued the “Guiding Opinions on Improving the Rural Living Environment,” specifying that “with village level environmental rectification as the focal point, comprehensively elevate the quality of the rural living environment” (19). In 2018, within the context of rural revitalization, China promulgated the “Three-Year Action Plan for the Improvement of the Rural Living Environment,” delineating three core tasks: “conducting the treatment of toilet waste, propelling the treatment of rural domestic garbage, and phasedly promoting the treatment of rural domestic sewage” (20).

Implementing the three-year improvement campaign has led to a remarkable improvement in the rural living environment in China. Moreover, it has generated a wealth of experience in rural living environment improvement, providing invaluable insights for implementing the “Five Year Action Plan for the Enhancement and Upgrade of the Rural Living Environment” proposed in the No. 1 Central Document of 2021 (21). Simultaneously, it helps subsequent policymakers establish and refine the long term governance mechanism for the rural living environment.

Spurred by the encouragement and support of the Chinese central government, local provinces and cities have progressively started to introduce rural environmental sanitation governance policies. As of June 6th, 2025, a cumulative total of 24 rural environmental governance policies have been issued by 7 cities across China. The policy content typically includes rural infrastructure construction, environmental governance measures, domestic garbage disposal, domestic sewage treatment, toilet waste treatment, agricultural non-point source pollution control, the optimization and preservation of village appearances, legal liabilities, and other related aspects. The comprehensiveness of the content vividly reflects the Chinese government’s unwavering resolve to promote the enhancement of the rural environmental sanitation governance standard.

### 3.2 Data sources and research samples

This study centers primarily on seven major cities in China where rural environmental governance policies have been put into practice. To ensure the recall rate of the selected research samples when collecting local policy texts, we employ the following retrieval strategy: Firstly, we conduct a literature review by accessing Chinese and foreign databases, acquire relevant literature, and extract and summarize local rural environmental governance policy texts therefrom. Secondly, we directly access relevant policy texts via online platforms such as the Chinese government’s information disclosure portal and official websites. Moreover, we utilize professional legal databases such as “Peking University Treasure” to search for relevant legal policy texts. Through this approach, a total of 24 rural environmental sanitation governance policies were retrieved (as of June 6, 2025) (22).

We minimize the interference from invalid and redundant text to ensure the research samples’ representativeness, authority, and relevance. This, in turn, helps enhance the precision of the research samples. While screening local rural environmental governance policy texts, we adhere to timeliness, relevance, effectiveness, and non-duplication principles. Firstly, by the timeliness principle, we rule out obsolete versions of legal policy texts and use the latest revised version as the research sample. Secondly, based on the relevance principle, we strictly limit the search terms to the semantic scope of highly relevant research topics such as “rural environment,” “environmental sanitation,” and “environmental governance.” According to the effectiveness principle, the study excludes all temporary government work documents such as social solicitation drafts, letters, and approvals. Fourthly, we eliminate similar texts based on the non-duplication principle. By following these principles, 7 representative policy documents were chosen as the research samples for this study, covering the period from 2018 to 2023. The detailed information is presented in Table 1.

**Table 1:** Rural environmental sanitation governance policies in China.

Code	Policy name	Release agency	Effective date
P1	*Harbin City Rural Environmental Sanitation Regulations*	The Standing Committee of the Harbin Municipal People’s Congress	2023.06
P2	*Jiujiang City Environmental Health Management Regulations*	The Standing Committee of the Jiujiang Municipal People’s Congress	2021.11
P3	*Linfen City Rural Environmental Health Comprehensive Management and Promotion Regulations*	The Standing Committee of the Linfen Municipal People’s Congress	2019.12
P4	*Yuncheng City Rural Environmental Management Regulations*	The Standing Committee of the People’s Congress of Yuncheng City	2019.03
P5	*Changchun City Rural Environmental Management Regulations*	The Standing Committee of the Changchun Municipal People’s Congress	2019.11
P6	*Turpan City Urban and Rural Environmental Sanitation Management Regulations*	The Standing Committee of the Turpan Municipal People’s Congress	2021.11
P7	*Yinchuan City Rural Environmental Protection Regulations*	The Standing Committee of the People’s Congress of Yinchuan City	2018.09

To enhance the transparency and reproducibility of our research, it is essential to further elaborate on the reasons for selecting 7 representative policies from the 24 retrieved ones. The selection of 7 policies is based on multiple considerations. Geographically, these 7 policies cover different regions of China, including the eastern, central, and western regions. This geographical diversity ensures that policies from areas with other economic development levels, population densities, and environmental conditions are included, allowing for a more comprehensive understanding of rural environmental governance policies across the country. Regarding policy types, the 7 policies represent different aspects of rural environmental sanitation governance, such as waste management, water pollution control, and ecological restoration. This variety enables us to explore the multifaceted nature of rural environmental policies.

### 3.3 Methods of analysis

This study employed the PMC index model to quantitatively evaluate the content of China’s rural environmental sanitation governance policies. The PMC index model, also known as the “Policy Consistency Index Model,” originated from the “Omnia Mobilis” hypothesis proposed by Ruize (23). It measures the design quality and validity of multiple policy samples through a multi-indicator system. Specifically, an evaluation system is formed with “9 primary variables + several secondary variables.” Subsequently, the cumulative effects of each secondary variable on the change of the overall sample are calculated and analyzed. Finally, a three-dimensional visual spatial view is applied to intuitively and vividly present the advantages and disadvantages of the policies.

#### 3.3.1 Construction of the PMC-index model

**Figure 1:**
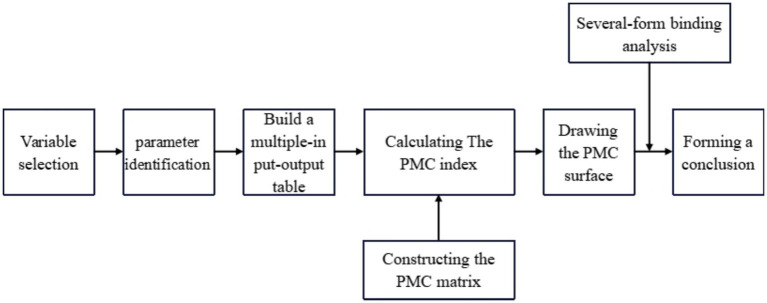
Construction framework of the PMC-Index model.

PMC index models follow the following application steps and tool framework (see Figure 1).

#### 3.3.2 Classification of variables and identification of parameters

This study employs the ROST Content Mining System 6 (ROSTCM6, developed by Wuhan University, Hubei, China) tool to conduct in-depth mining and word-frequency statistics on 7 policy texts. The selection of nine primary variables and 37 sub-variables is grounded in a hybrid theoretical framework that integrates policy analysis theory, Sustainable Development Goals (SDGs), and China’s rural governance practices.

##### 3.3.2.1 Theoretical foundation for primary variables

X_1_ (Policy Nature) and X_2_ (Policy Timeliness) are derived from policy cycle theory (24), which emphasizes the importance of feedback mechanisms and phased planning in policy sustainability. For example, the inclusion of sub-variables such as “feedback” (X_1:3_) and “medium-term planning” (X_2:2_) reflects the need for adaptive governance. X_3_ (Policy Perspective) and X_6_ (Policy Field) align with the multi-dimensional policy analysis framework (25), which categorizes policies into macro/micro perspectives and cross-sectoral domains (e.g., politics, economy, ecology). This ensures coverage of both strategic vision (e.g., “macroscopic view,” X_3:1_) and operational details (e.g., “microscopic view,” X_3:2_). X_4_ (Policy Type) and X_8_ (Supporting Measures) are informed by institutional design theory (26), which highlights the role of mandatory/non-mandatory instruments and resource guarantees (e.g., funding, legal regulations) in policy implementation. X_5_ (Policy Target) and X_7_ (Policy Function) draw from stakeholder theory and rural development theory (27), emphasizing the inclusion of diverse actors (e.g., “educational institutions,” X_5:5_) and multifunctional goals (e.g., “agricultural development,” X_7:5_). X_9_ (Policy Quality) synthesizes policy legitimacy theory (28), focusing on the sufficiency of policy basis (X_9:1_) and clarity of goals (X_9:2_) to ensure normative validity.

##### 3.3.2.2 Coverage of China’s rural environmental governance priorities

The 37 sub-variables were specifically tailored to address key challenges in China’s rural sanitation governance, as identified in national policy documents such as the Three-Year Action Plan for Rural Living Environment Improvement (2018) and the Five-Year Action Plan for Rural Revitalization (2021). For example, Sub-variables under X_5_ (Policy Target; e.g., “grass-roots organizations,” X_5:3_) and X_8_ (Supporting Measures; e.g., “legal regulation,” X_8:2_) directly respond to the central government’s emphasis on grassroots participation and institutionalization. Environmental protection-related sub-variables (e.g., “sewage treatment,” X_6:4_) and economic development links (e.g., “agricultural development,” X_7:5_) reflect the dual goals of ecological conservation and rural revitalization stipulated in China’s rural policy framework (29).

##### 3.3.2.3 Validation through policy text analysis

This article employed the ROSTCM6 text-mining software to conduct in-depth mining and word-frequency statistics on seven policy texts. The preprocessing steps for text mining included: (1) Text cleaning, namely removing irrelevant metadata (e.g., policy titles, signatures) and standardizing terminology (e.g., unifying “garbage disposal” and “waste management” into a single term); (2) Stopword elimination, that is filtering out common Chinese particles (“should,” “must”), pronouns, and prepositions that carry no semantic weight for policy analysis; (3) Part-of-speech (POS) tagging, which means using the software’s built-in POS parser to identify nouns, verbs, and adjectives relevant to policy components (e.g., “target,” “measure,” “timeline”).

After preprocessing, the software generated a word-frequency matrix for the top 40 nouns and action verbs (see Table 2), which were then mapped to initial evaluation indicators. For example, high-frequency terms such as “environment” (420 times) and “Government” (187 times) directly informed the inclusion of variables such as X_3_ (Policy Perspective) and X_4_ (Policy Type). An evaluation system framework covering nine primary variables and 37 sub-variables was thus established (see Table 3). Quantitative variables were derived by assigning binary values (1/0) to sub-variables based on the policy text’s presence/absence of specific terms or phrases. For instance, the sub-variable X_5:5_ (educational institutions’ involvement) was coded as “1″ if the policy mentioned “educational institutions” in the context of environmental education.

**Table 2:** Hot words and their frequencies in sample policies.

Vocabulary	Frequency	Vocabulary	Frequency	Vocabulary	Frequency	Vocabulary	Frequency
Environment	420	Unit	103	Organization	61	Agriculture	38
Rural	321	Governance	99	Waste	58	Timely	37
Hygiene	294	Villagers	88	Public	55	Represent	37
Garbage	246	Sewage	81	Area	55	Members	36
Government	187	Protection	74	Poultry	53	Office	34
Management	150	Responsibility	73	Fines	47	Correction	34
Department	141	Supervisor	71	Planning	46	Society	33
Facility	120	Committee	66	Supervision	46	Toilet	33
Construction	109	Behavior	64	Pollution	45	Law	31
Regulation	108	Collection	64	Street	39	Transport	31

**Table 3:** Quantity indicators and parameter settings.

Primary variable	Secondary variable	Evaluation scale	Evaluation parameter
X_1_ policy nature	X_1:1_ Prediction	Whether reflects the prediction?	X_1:1_–N[0,1]
	X_1:2_ Suggestion	Whether reflects the Suggestion?	X_1:2_–N[0,1]
	X_1:3_ Feedback	Whether reflects the Feedback?	X_1:3_–N[0,1]
	X_1:4_ Supervision	Whether reflects the supervision?	X_1:4_–N[0,1]
	X_1:5_ Description	Whether reflects the description?	X_1:5_–N[0,1]
	X_1:6_ Guidance	Whether reflects the guidance?	X_1:6_–N[0,1]
X_2_ policy timeliness	X_2:1_ Long term	Whether it involves longer than 5 years?	X_2:1_–N[0,1]
	X_2:2_ Medium term	Whether it involves 3–5 years?	X_2:2_–N[0,1]
	X_2:3_ Short term	Whether it involves 1–3 years?	X_2:3_–N[0,1]
X_3_ policy perspective	X_3:1_ Macroscopic view	Whether it formulated from a macro perspective?	X_3:1_–N[0,1]
	X_3:2_ Microscopic view	Whether it formulated from a micro perspective?	X_3:2_–N[0,1]
X_4_ policy type	X_4:1_ Mandatory	Is the policy a mandatory type?	X_4:1_–N[0,1]
	X_4:2_Non-mandatory	Is the policy a non-mandatory?	X_4:2_–N[0,1]
X_5_ policy target	X_5:1_ Superior government	Whether the object involves the superior government?	X_5:1_–N[0,1]
	X_5:2_ Grassroots public sector	Whether the action object involves the grassroots public sector?	X_5:2_–N[0,1]
	X_5:3_ Grass-roots organizations	Whether the object of action involves the grass-roots organizations?	X_5:3_–N[0,1]
	X_5:4_ Regulatory authorities	Whether the acting object involves the regulatory authorities?	X_5:4_–N[0,1]
	X_5:5_ Educational institutions	Whether the object involves the educational institutions?	X_5:5_–N[0,1]
X_6_ policy field	X_6:1_ Politics	Whether politics is involved?	X_6:1_–N[0,1]
	X_6:2_ Economy	Whether it involves the economy?	X_6:2_–N[0,1]
	X_6:3_ Society	Whether it involves society?	X_6:3_–N[0,1]
	X_6:4_ Ecology	Whether it involves ecology?	X_6:4_–N[0,1]
	X_6:5_ Technology	Whether it involves technology?	X_6:5_–N[0,1]
X_7_ policy function	X_7:1_ Bettering the living environment	Whether it includes the content of optimizing the living environment?	X_7:1_–N[0,1]
	X_7:2_Popularizing the concept of environmental protection	Whether it includes the content of Popularizing the concept of environmental protection?	X_7:2_–N[0,1]
	X_7:3_ Promoting cooperation in environmental protection	Whether it includes the content of promoting cooperation in environmental protection?	X_7:3_–N[0,1]
	X_7:4_ Forming rural culture	Whether it includes the content of forming rural culture?	X_7:4_–N[0,1]
	X_7:5_ Promoting agricultural development	Whether it includes the content of promoting agricultural development?	X_7:5_–N[0,1]
	X_7:6_ Improving rural governance	Whether it includes the content of improving rural governance?	X_7:6_–N[0,1]
X_8_ supporting measures	X_8:1_ Funding incentives	Whether there is a financial incentive as a guarantee?	X_8:1_–N[0,1]
	X_8:2_ Legal regulation	Whether there is a legal regulation as the guarantee?	X_8:2_–N[0,1]
	X_8:3_ Organization guarantee	Whether there is an organized guarantee for the guarantee?	X_8:3_–N[0,1]
	X_8:4_ Resource planning	Whether there is a resource planning for the guarantee?	X_8:4_–N[0,1]
X_9_ policy quality	X_9:1_ With sufficient basis	Whether the policy text is well-grounded?	X_9:1_–N[0,1]
	X_9:2_ With specific goals	Whether the policy text is clear in purpose?	X_9:2_–N[0,1]
	X_9:3_Setting forth a sound plan	Whether the policy text is a scientific program?	X_9:3_–N[0,1]
	X_9:4_With detailed content	Whether the policy text is detailed?	X_9:4_–N[0,1]

Based on the above analysis, this study integrates the characteristics of China’s rural environmental sanitation governance policies and refers to relevant scholarly research on this topic. It establishes an evaluation index system for China’s rural environmental sanitation governance policies, comprising nine primary variables and 37 sub-variables that comprehensively cover the content of policy documents (Table 3).

#### 3.3.3 Construct a multi-input–output table

The multi-input–output table features an application structure tailored to database analysis and provides ample storage space for the data to conduct in-depth analysis of various variables. It enables the indicators of the PMC index model (a model used to evaluate policy-related aspects) to reflect the overall policy validity. This paper uses it as the analysis framework for measuring rural environmental sanitation governance policies. The main variables, which are composed of multiple sub-variables, play a crucial role in this analysis. For details regarding the specific settings of these variables, see Table 4.

**Table 4:** Multi-input–output table.

Variable	Primary variable								
	X_1_	X_2_	X_3_	X_4_	X_5_	X_6_	X_7_	X_8_	X_9_
Secondary variable	X_1:1_	X_2:1_	X_3:1_	X_4:1_	X_5:1_	X_6:1_	X_7:1_	X_8:1_	X_9:1_
	X_1:2_	X_2:2_	X_3:2_	X_4:2_	X_5:2_	X_6:2_	X_7:2_	X_8:2_	X_9:2_
	X_1:3_	X_2:3_			X_5:3_	X_6:3_	X_7:3_	X_8:3_	X_9:3_
	X_1:4_				X_5:4_	X_6:4_	X_7:4_	X_8:4_	X_9:4_
	X_1:5_				X_5:5_	X_6:5_	X_7:5_		
	X_1:6_						X_7:6_		

#### 3.3.4 Measurement of the PMC-index

This paper follows the following steps to calculate the PMC index of each sample to be tested: first, the 9 main variables and 37 sub variables contained in the evaluation system of China’s rural environmental sanitation governance policy are placed in the multi input–output table in turn; Secondly, make full use of text mining tools and record and output the specific values of different sub variables under the same main variable according to the following Equations 1, 2; Then, the specific values of each main variable are calculated through Equation 3. Finally, according to Equation 4, the PMC indexes of the 12 rural environmental sanitation governance policies in China that need to be evaluated are calculated respectively, which can be used as the basis for the subsequent direct judgment of the policy effect.


(1) G:N[0,1]

(2) G={GR:[0,1]}

(3) $$G_{i}=(\sum_{j=1}^{n}\frac{G_{\mathrm{ij}}}{T(G_{\mathrm{ij}})})i=1,2,\ldots 9$$

(i is the primary variable, j is the sub variable, t is the number of sub variables, the same below).


(4) $$\mathrm{PMC}-\text{Index}=[\begin{array}{l} G_{1}\sum_{j=1}^{6}\frac{G_{1j}}{6}+G_{2}\sum_{j=1}^{3}\frac{G_{2j}}{3}+G_{3}\sum_{j=1}^{2}\frac{G_{3j}}{2}\\ G_{4}\sum_{j=1}^{2}\frac{G_{4j}}{6}+G_{5}\sum_{j=1}^{5}\frac{G_{5j}}{5}+G_{6}\sum_{j=1}^{5}\frac{G_{6j}}{5}\\ G_{7}\sum_{j=1}^{6}\frac{G_{7j}}{6}+G_{8}\sum_{j=1}^{4}\frac{G_{8j}}{4}+G_{9}\sum_{j=1}^{4}\frac{G_{9j}}{4} \end{array}]$$

Upon calculating the PMC index for all samples under test, the researchers compare the derived PMC index with the grading criteria to ascertain the corresponding rating. Grounded in the rating-score classification table put forward by Estrada, this paper revises the existing standard to formulate the classification criterion for China’s rural environmental sanitation governance policies. The scoring criterion adopts a complete-mark system of 9 points, which is categorized into five grades:

- In the event that the PMC index score is lower than 2.99, it serves as an indication that the policy consistency is abysmally poor and the policy-making effect is substandard.
- When the score lies within the range of 3.00 to 4.99, it implies that the policy consistency is moderate and the policy-making effect is tolerable.
- If the score is between 5.00 and 6.99, it demonstrates that the policy consistency is commendable and the policy-making effect is favorable.
- When the score ranges from 7.00 to 8.99, it reveals that the policy consistency is highly notable and the policy-making effect is outstanding.
- In the case where the score attains 9, the policy effect is flawless, and the most rational state has been achieved.

#### 3.3.5 Construction of the PMC-surface

To enhance the overall visualization effect of the PMC matrix, this paper generates the PMC surface. The generation of the PMC surface follows a specific mathematical model, as shown in Equation 5. The PMC surface, composed of the results of the nine principal variables in Table 4, assumes a three dimensional configuration. Since the PMC matrix has the same number of rows and columns, the generated PMC surface is symmetrical. Furthermore, it can reflect the formulation effect of the evaluated policy through the surface’s concavity, convexity, and position.


(5) $$\mathrm{PMC}-\text{Surface}=[\begin{array}{l} G_{1}\;\;G_{2}\;\;G_{3}\\ G_{4}\;\;G_{5}\;\;G_{6}\\ G_{7}\;\;G_{8}\;\;G_{9} \end{array}]$$

## 4 Results and analysis

### 4.1 Empirical results

**Figure 2:**
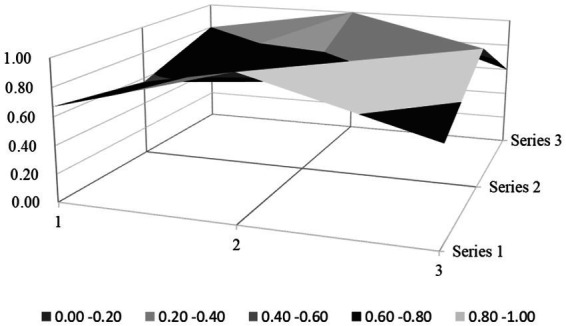
PMC-surface chart of P1 (Good).

**Figure 3:**
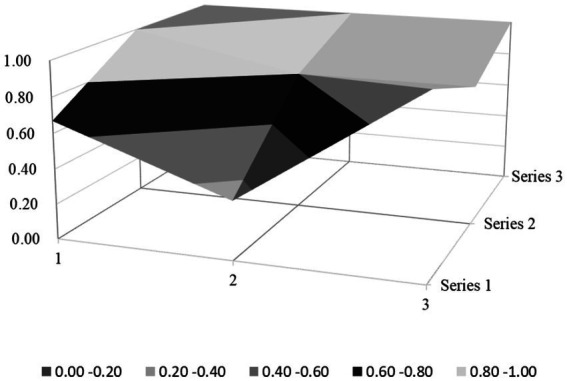
PMC-surface chart of P2 (Excellent).

**Figure 4:**
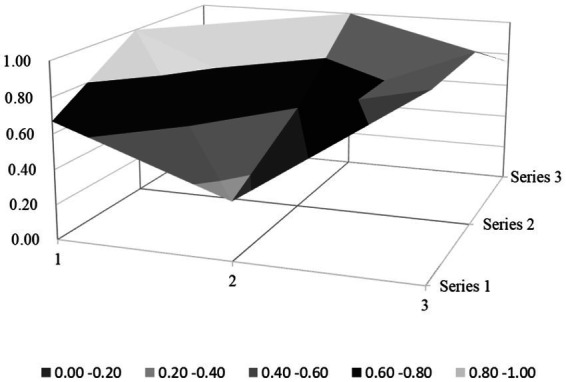
PMC-surface chart of P3 (Good).

**Figure 5:**
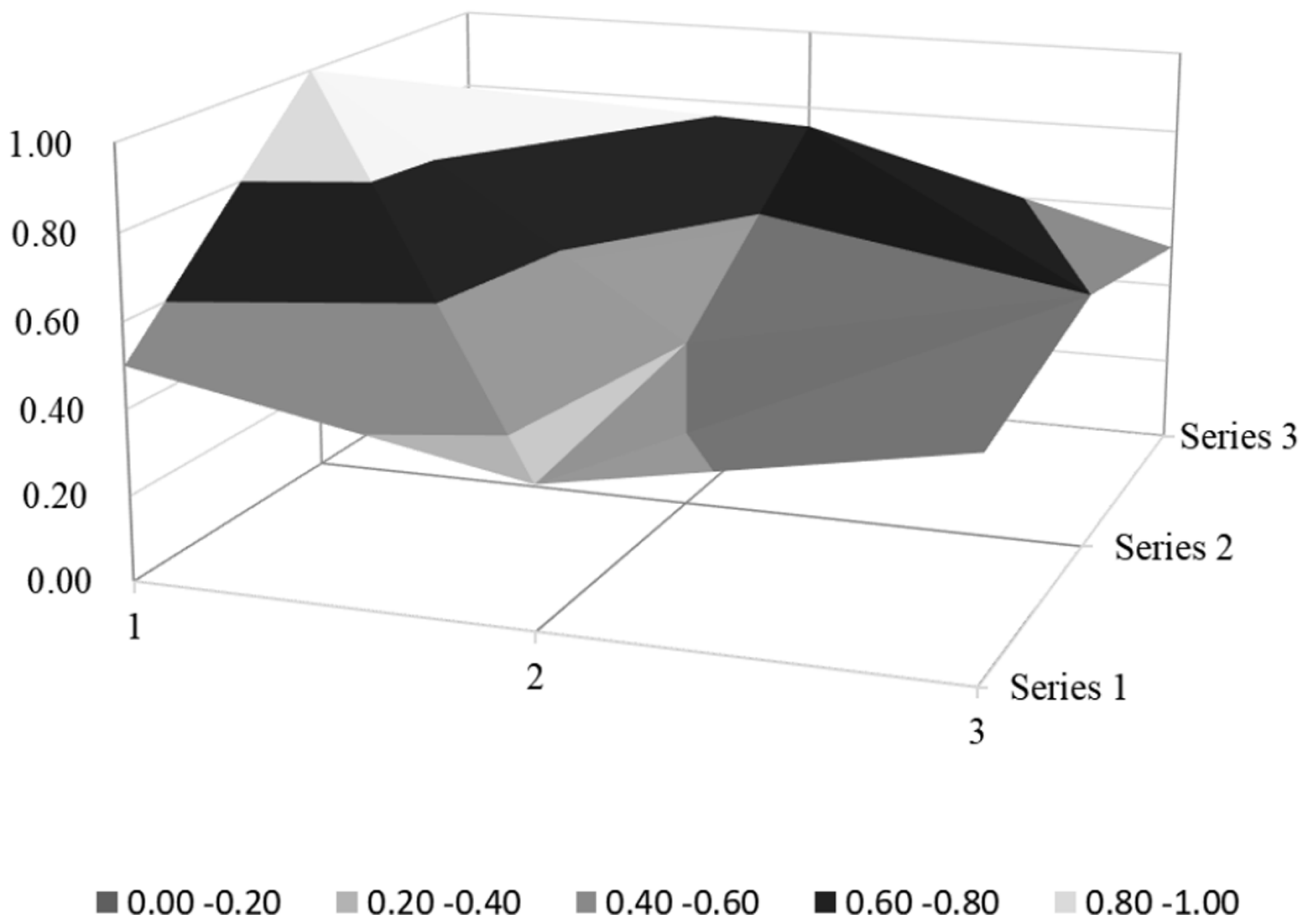
PMC-surface chart of P4 (Unsatisfactory).

**Figure 6:**
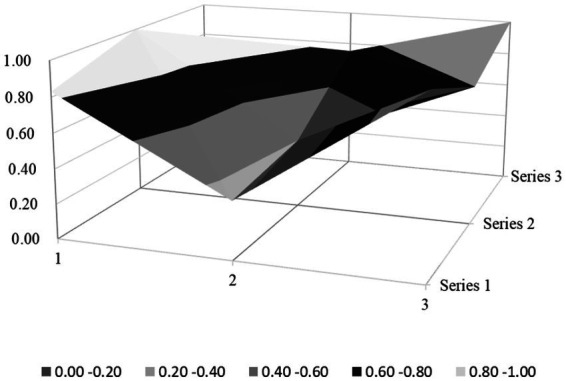
PMC-surface chart of P5 (Good).

**Figure 7:**
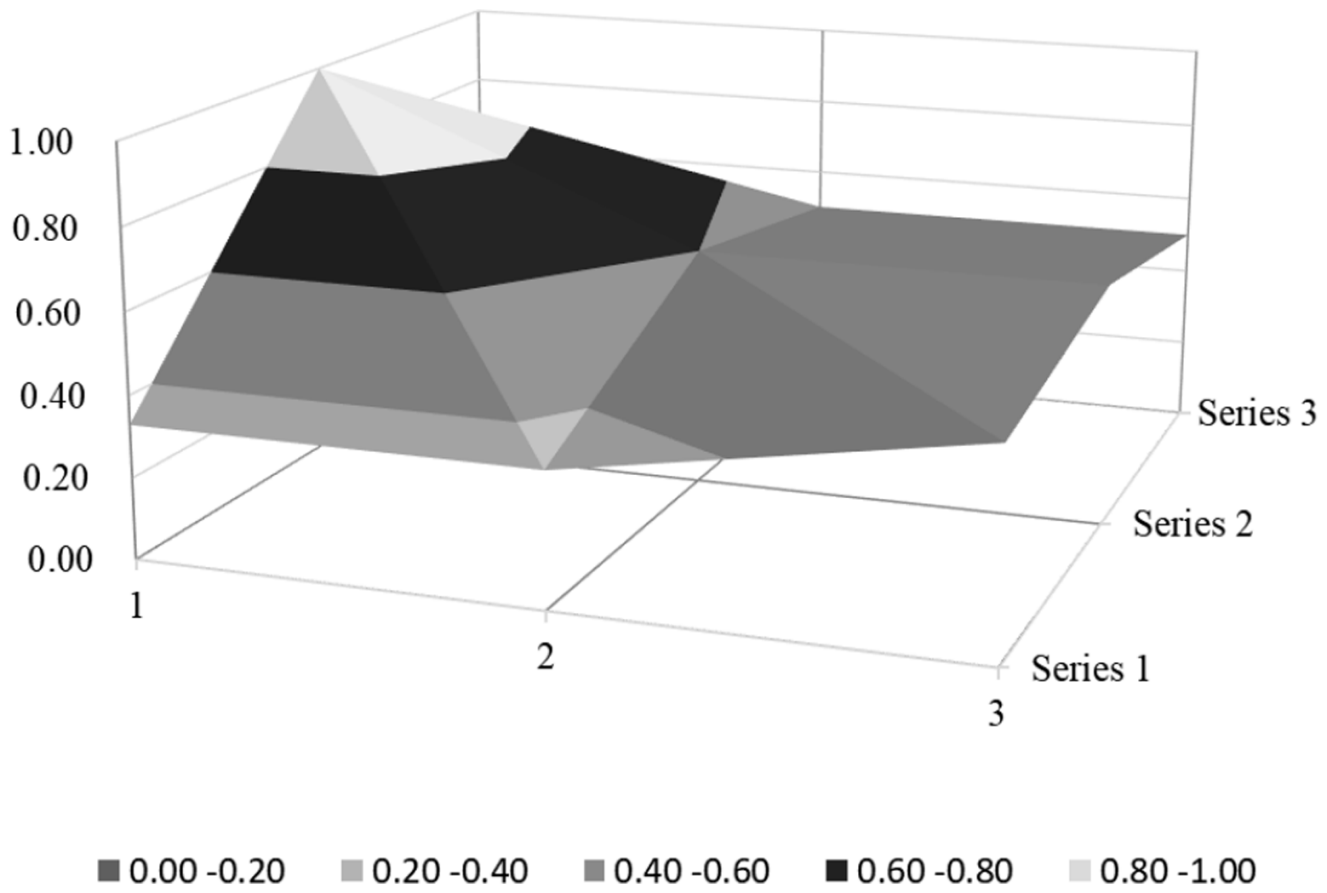
PMC-surface chart of P6 (Unsatisfactory).

**Figure 8:**
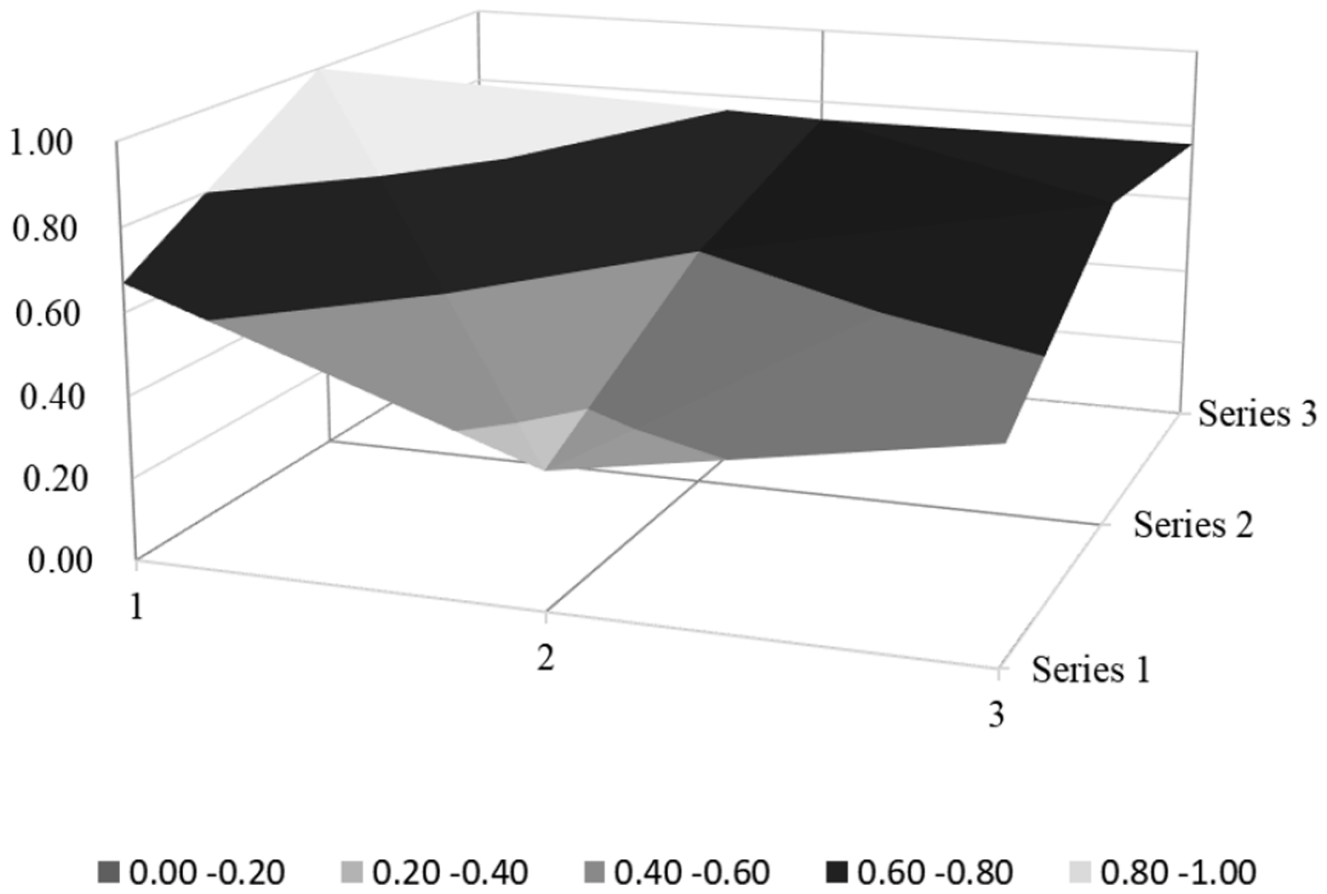
PMC-surface chart of P7 (Good).

Based on the PMC index evaluation model constructed above, this paper obtains multiple input–output tables (see Table 5) of seven policy samples through text mining technology. On this basis, this paper calculates the PMC index, rounded to two decimal places, and the ranking of each policy (see Table 6). Moreover, based on the PMC matrix scores of each policy, the PMC surface plots in Figures 2–8 can be drawn. Here, (1, 2, 3) serve as the horizontal axis, and (Series 1, Series 2, Series 3) as the vertical axis. Thus, (1, Series 1) represents G_1_, (2, Series 1) represents G_2_, (3, Series 1) represents G_3_, and so on. Different shades of color distinguish different score levels, and varying degrees of depression or elevation indicate different score magnitudes (the elevated parts denote higher scores, while the depressed parts indicate lower scores). These operations aim to comprehensively evaluate the policy-making effects of the 7 policy samples.

**Table 5:** Multi-input–output table of seven policy samples.

Primary variable	Secondary variable	P1	P2	P3	P4	P5	P6	P7
X_1_	X_1:1_	0	1	1	0	1	0	0
	X_1:2_	1	1	1	1	1	1	1
	X_1:3_	0	0	0	0	0	0	0
	X_1:4_	1	1	1	1	1	1	1
	X_1:5_	0	0	0	0	1	0	1
	X_1:6_	1	1	1	1	1	0	1
X_2_	X_2:1_	1	1	1	1	1	1	1
	X_2:2_	0	0	0	0	0	0	0
	X_2:3_	0	0	0	0	0	0	0
X_3_	X_3:1_	1	1	1	0	1	0	0
	X_3:2_	1	1	1	1	1	1	1
X_4_	X_4:1_	1	1	1	1	1	1	1
	X_4:2_	1	1	1	1	1	1	1
X_5_	X_5:1_	0	1	0	0	0	0	1
	X_5:2_	1	1	1	1	1	1	1
	X_5:3_	1	1	1	1	1	1	1
	X_5:4_	0	1	0	0	0	0	0
	X_5:5_	1	0	1	0	0	1	0
X_6_	X_6:1_	1	0	1	0	0	0	0
	X_6:2_	1	1	1	0	1	0	1
	X_6:3_	1	1	1	1	1	1	1
	X_6:4_	1	1	1	1	1	1	1
	X_6:5_	1	1	1	1	1	1	1
X_7_	X_7:1_	1	1	1	1	1	1	1
	X_7:2_	0	1	0	0	0	0	0
	X_7:3_	0	1	0	0	0	0	0
	X_7:4_	0	1	0	1	0	0	0
	X_7:5_	0	1	0	0	0	0	1
	X_7:6_	1	1	1	0	1	0	0
X_8_	X_8:1_	1	1	1	1	0	0	0
	X_8:2_	1	1	1	1	1	1	1
	X_8:3_	1	1	1	1	1	1	1
	X_8:4_	1	1	1	0	1	0	1
X_9_	X_9:1_	1	1	1	1	1	1	1
	X_9:2_	1	1	1	1	1	1	1
	X_9:3_	0	1	0	0	1	0	0
	X_9:4_	0	1	1	0	1	0	1

**Table 6:** PMC-index and level of seven policies.

Primary variable	P1	P2	P3	P4	P5	P6	P7	Mean value
X_1_	0.50	0.67	0.67	0.50	0.83	0.33	0.67	0.60
X_2_	0.33	0.33	0.33	0.33	0.33	0.33	0.33	0.33
X_3_	1.00	1.00	1.00	0.50	1.00	0.50	0.50	0.79
X_4_	1.00	1.00	1.00	1.00	1.00	1.00	1.00	1.00
X_5_	0.60	0.80	0.60	0.40	0.40	0.60	0.60	0.57
X_6_	1.00	0.80	1.00	0.60	0.80	0.60	0.80	0.80
X_7_	0.33	1.00	0.33	0.33	0.33	0.17	0.33	0.40
X_8_	1.00	1.00	1.00	0.75	0.75	0.50	0.75	0.82
X_9_	0.50	1.00	0.75	0.50	1.00	0.50	0.75	0.71
PMC-index	6.26	7.6	6.68	4.91	6.44	4.53	5.73	6.02
Rank	4	1	2	6	3	7	5	
Grade	C	B	C	D	C	D	C	

### 4.2 Holistic analysis

**Figure 9:**
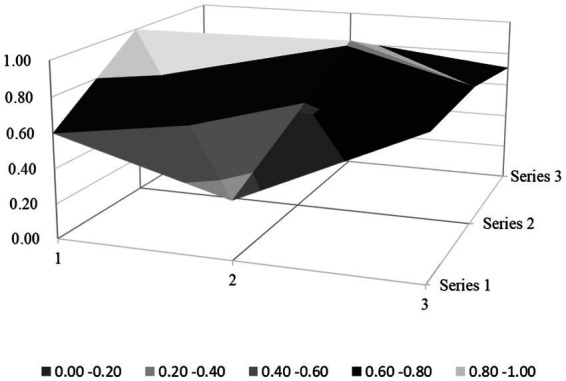
PMC-surface chart of mean value (Good).

From the overall performance of various policies in Table 6, the average PMC index of the 7 policies is 6.02, indicating that the integrity and consistency of the existing policies are generally good. Based on the PMC index, the 7 policies are ranked as P2 > P3 > P5 > P1 > P7 > P4 > P6. According to the grading criteria, among the 7 policy samples, 1 policy is rated as excellent, 4 are good, and 2 are unsatisfactory. The overall quality of the 7 policies is relatively good, and the policy content has a certain degree of integrity and rationality. However, there are still several policies of poor quality, suggesting that there is still much room for improvement in the content design of the sample policies. In addition, from the basic shape of the PMC surface diagram in Figure 9, the average PMC surface diagrams of the 7 policies are relatively smooth as a whole, indicating that the formulation effects of the 7 policy samples are good, and the internal structures of each policy are relatively reasonable. Regarding the distribution of policy release times, the introduction times of the 7 policies on rural environmental sanitation governance in China span an extensive range and are scattered. It can be found that there is no obvious overall correlation between the PMC index scores of the sample policies and their introduction times. This indicates that the sample policies introduced later did not fully absorb the experience of the previously formulated policies, and there is a lack of necessary interaction and communication between the policies of different cities.

Analysis of primary variable scores reveals consistently higher averages for X_3_ (Policy Perspective), X_4_ (Policy Type), X_6_ (Policy Field), X_8_ (Supporting Measures), and X_9_ (Policy Quality) are relatively high, indicating that policymakers pay more attention to these dimensions when formulating relevant policies and incorporate them into the key content of the policies. However, the lower scores of the main variables X_1_ (Policy Nature), X_2_ (Policy Timeliness), X_5_ (Policy Target), and X_7_ (Policy Function) can be attributed to structural flaws in policy texts and gaps in implementation logic.

For X_1_ (Policy Nature), the sample policies generally lack dynamic feedback mechanisms, as evidenced by the X_1:3_ “feedback” indicator scoring 0 across all 7 policies prevents policies from adapting to farmer feedback or environmental data changes during implementation. For example, the policies in Turpan (P6) and Yuncheng (P4) fail to include “annual policy effectiveness evaluation” clauses and directly adopt urban centralized waste treatment models without considering the scattered nature of mountain villages and high transportation costs, resulting in most of the facilities being idle. Additionally, vague definitions of core concepts, such as the average score of only 0.57 for the X_1:5_ “description” indicator, mean that “rural environmental sanitation” does not explicitly include agricultural non-point source pollution control, leading grassroots implementation to focus solely on domestic waste cleaning and neglecting the recycling of pesticide packaging waste.

Regarding X_2_ (Policy Timeliness), while all policy texts set long-term goals (with the X_2:1_ “long-term” indicator fully scored as 1), they lack mid-term and short-term stage divisions (with X_2:2_ “mid-term” and X_2:3_ “short-term” indicators both at 0), resulting in a lack of trackable quantitative milestones. Take Harbin (P1) as an example, its goal of “full coverage of sewage pipelines by 2025” is not broken down into annual tasks, increasing the risk of implementation delays. This design flaw of “emphasizing outcomes over processes” causes local governments to lack phased assessment pressures, making delays or formalistic implementation more likely.

X₅ reveals constrained stakeholder inclusivity, as only Jiujiang (P2) explicitly incorporates educational institutions (with the X_5:5_ “educational institutions” indicator at 1). At the same time, the other six policies do not mention social forces. Empirical data shows that the average awareness rate of household waste sorting in areas without educational institutions promotion is 30%, compared to 73% in Jiujiang due to the mechanism of “2 environmental practice classes per semester in primary and secondary educational institutions” (30). At the same time, some policies have vague descriptions of government responsibilities, such as Changchun (P5), only mentioning that “grassroots public departments are responsible for governance” without clarifying coordination mechanisms among departments, which leads to overburdened village-level organizations and low garbage collection frequency.

X₇ demonstrates functional narrowness, the policy functions are highly concentrated on “improving the living environment” (with the X_7:1_ indicator fully scored as 1). Still, the synergistic value of culture and economy is ignored. 6 policies do not mention “rural ecological culture inheritance” (X_7:4_), such as Yinchuan (P7)'s toilet renovation project, which does not incorporate local folklore into facility design, leading most farmers to revert to using traditional toilets due to a lack of cultural identity. Only Jiujiang (P2) sets “garbage resource utilization subsidies” (X_7:5_ = 1), while other regions fail to integrate environmental sanitation governance with rural tourism or circular agriculture. For example, Linfen (P3)'s straw burning prohibition policy lacks supporting clauses for cooperation with biomass energy enterprises, resulting in a 45% rebound rate of illegal burning by farmers.

### 4.3 Scoring situation of each policy

To further clarify the characteristics of China’s rural environmental sanitation governance policies and the problems existing in their content design, this paper conducts a detailed analysis of the evaluation of 7 policy samples using multi-input–output tables and PMC index scores (see Tables 5, 6).

#### 4.3.1 Excellent level policy

P2 was released relatively late, and the residents enjoyed a relatively high average income. Therefore, there was a solid material foundation for its policy-making. The scores of the main variables X_3_ (Policy Perspective), X_4_ (Policy Type), X_7_ (Policy Function), X_8_ (Supporting Measures), and X_9_ (Policy Quality) of P2 were all full marks, and the scores of all main variables were higher than the average. This indicated the policy was nearly perfect, with outstanding formulation effects, excellent policy integrity, and consistency.

Regrettably, P2 still lost points regarding four dimensions: X_1_ (Policy Nature), X2 (Policy Timeliness), X_5_ (Policy Target), and X_6_ (Policy Field), leaving room for further improvement. In X_1_ (Policy Nature), P2 lacked the necessary policy feedback mechanism and conceptual description, meaning that its interactive ability, dynamic adjustment ability, and language accessibility were poor. In X_2_ (Policy Timeliness), P2 only focused on establishing long-term mechanisms while neglecting short- and medium-term planning, which was not conducive to the gradual implementation of the policy. In X_5_ (Policy Target), the content of P2 only covered the Superior government, the Grassroots public sector, the grassroots organizations, and the Regulatory authorities, ignoring the role of educational institutions in publicity and education. In X_6_ (Policy Field), P2 overlooked the value of China’s rural environmental sanitation governance policy in achieving political goals.

#### 4.3.2 Good level policy

P1, P3, P5, and P7 policy ratings are classified as “good.” Overall, the average Policy-Making Capacity (PMC) index score for these well-rated policies stands at 6.46. Notably, in dimensions X_3_ (Policy Perspective), X_4_ (Policy Type), X_6_ (Policy Field), X_8_ (Supporting Measures), and X_9_ (Policy Quality), these policies have achieved relatively high scores, with an average value exceeding 0.70. However, the average scores in X_1_ (Policy Nature), X_2_ (Policy Timeliness), X_5_ (Policy Target), and X_7_ (Policy Function) are comparatively low, thereby necessitating focused attention and improvement efforts.

When examined in detail, in the context of X_1_ (Policy Nature), the three policy texts rated as “good” commonly exhibit a lack of content regarding establishing a feedback mechanism. Moreover, they fail to offer essential explanations for important concept terms likely to cause ambiguity. In X_2_ (Policy Timeliness), every policy rated “good” does not delineate the time-limit structure and phased development plans. Instead, they merely vaguely define long-term development goals and plans, while neglecting short- and medium-term objectives and plans. In X_5_ (Policy Target), all the policies with a “good” rating do not fully consider the functional implementation of the superior government and regulatory authorities. These two entities play pivotal roles in China’s rural environmental sanitation governance. In X_7_ (Policy Function), all policies rated as “good” do not emphasize their roles in popularizing environmental protection concepts, promoting collaborative environmental protection efforts, fostering rural culture, and elevating agricultural development.

#### 4.3.3 Unsatisfactory level policy

P4 and P6 are rated as unsatisfactory. The Policy-Making Capacity (PMC) index scores for these policies all fall below average. Meanwhile, except for X_2_ (Policy Timeliness) and X_4_ (Policy Type), the average scores of the PMC index on other main variables also lag behind the overall average score. This indicates that the overall quality of the policies rated as unsatisfactory is low, characterized by extremely poor consistency. Thus, policymakers should revise, adjust, and optimize the policy texts. However, on the whole, numerous similar issues exist among the unsatisfactory, good-rated, and excellent-rated policies. When viewed from the perspective of dynamic policy implementation, problems such as ambiguous policy timeliness definition, narrow policy field coverage, and inadequate policy functions are widespread. Simultaneously, a significant disparity remains between the unsatisfactory policies and the others mentioned above.

From the perspective of static policy texts, the two policies under consideration suffer from an insufficient formulation basis, unclear policy goals, unscientific implementation plans, and insufficiently detailed text content. These policies must be enhanced and rectified without delay; otherwise, they will impede the advancement of rural environmental sanitation governance in China.

### 4.4 PMC surface morphology and policy implications

The PMC surface diagrams (Figures 9–8) visually demonstrate policy performance through three-dimensional morphology, where protrusions represent high scores (practical design elements) and depressions indicate low scores (structural gaps). Based on the above scoring results, this analysis further systematically interprets their morphological differences to reveal the underlying policy implications.

#### 4.4.1 Morphological and policy analysis of excellent level policy

P2 (Jiujiang City, Excellent, PMC = 7.6) presents a “multi-peak platform” structure on its surface (Figure 3), with significant protrusions in the dimensions of X_3_ (Policy Perspective), X_4_ (Policy Type), X_7_ (Policy Function), X_8_ (Supporting Measures), and X_9_ (Policy Quality), while showing minor depressions in X_1_ (Policy Nature), X_2_ (Policy Timeliness), and X_5_ (Policy Targets).

Specifically, the key protrusion in the X_7_ (Policy Function) dimension—the highest peak on the surface—originates from its design of multi-stakeholder collaboration involving educational institutions (X_5:5_ = 1), enterprises, and communities. For instance, integrating “two environmental practice classes per semester in primary and secondary schools” increased household waste sorting awareness to 73%, significantly higher than the 30% rate in regions without educational engagement. Additionally, linking the improvement of rural infrastructure (X_7:5_ = 1) with rural tourism and cultural heritage preservation (X_7:4_ = 1) generates synergistic environmental, economic, and cultural effects. The gentle protrusion in the X_8_ (Supporting Measures) dimension (X_8:1_–X_8:4_ = 1) reflects a complete institutional framework, such as tax incentives for enterprises investing in sanitation infrastructure, which addresses the limitation of traditional single-government funding. However, the depressions in X_1_ (lack of feedback mechanisms) and X_2_ (absence of medium-and short-term planning) may hinder the policy’s long-term adaptability to evolving rural needs.

#### 4.4.2 Morphological and policy analysis of good level policy

The “Good” policy group (P1, P3, P5, P7) demonstrates moderate overall performance with PMC indices ranging from 5.73 to 6.68, reflecting structural strengths in macro-level design but persistent micro-level gaps that limit effectiveness. Their PMC surfaces exhibit asymmetrical protrusions in policy perspective, type, and field, countered by depressions in timeliness, stakeholder engagement, and functional diversity. The detailed analysis is as follows:

P1 (Harbin City, PMC = 6.26): macro-aligned but process-deficient. Its surface morphology (Figure 2) is characterized by concentrated protrusions in X_3_ (Policy Perspective; macro–micro balance, X_3:1_ = 1, X_3:2_ = 1) and distinct elevations in X_8_ (Supporting Measures; legal regulation, X_8:2_ = 1), yet exhibits pronounced depressions in X_2_ (Policy Timeliness; 0.33/1) and X_5_ (Policy Targets; 0.60/1). The policy aligns with policy cycle theory through its macro-level goal setting (e.g., “full sewage pipeline coverage by 2025”) but violates the theory’s emphasis on phased implementation by omitting mid/short-term milestones (X_2:2_ = 0, X_2:3_ = 0). While adhering to a multidimensional policy analysis framework by covering ecological (X_6:4_ = 1) and technical (X_6:5_ = 1) domains, it fails to operationalize stakeholder theory by excluding educational institutions (X_5:5_ = 0). Empirical evidence shows that the lack of annual task decomposition (e.g., quarterly pipeline-laying targets) caused project timeline delays, while vague interdepartmental coordination (X_5:4_ = 0) further led to misalignment in waste collection responsibilities, reducing service frequency in targeted villages.

P3 (Linfen City, PMC = 6.68): technically focused but stakeholder-narrow. Its surface morphology (Figure 4) features dual protrusions in X_3_ (Policy Perspective; macroscopic view, X_3:1_ = 1) and technological integration in X_6_ (Policy Field; X_6:5_ = 1), yet these advantages are offset by valley-like depressions in X_7_ (Policy Function; 0.33/1) and X_5_ (Policy Targets; 0.60/1). While the policy employs institutional design theory through mandatory policy tools (X_4:1_ = 1) and legal guarantees (X_8:2_ = 1), it lacks adaptive governance mechanisms (X_1:3_ = 0), thereby failing to address local variations in straw burning practices. Although aligning with rural development theory by promoting ecological technologies (e.g., biomass energy projects), it neglects social capital building by excluding educational institutions (X_5:5_ = 0) and community networks. Empirically, the sole focus on “improving living environments” (X_7:1_ = 1) without agricultural integration (X_7:5_ = 0) has led to an increase in illegal straw burning incidents, as farmers lack economic incentives to adopt biomass alternatives; meanwhile, the absence of “environmental stewardship” programs in schools (as in P2) has resulted in a lower waste-sorting compliance rate compared to cities with educational engagement.

P5 (Changchun City, PMC = 6.44): normative but participation-limited. Its surface morphology (Figure 6) shows stable protrusions in the X_9_ (Policy Quality) dimension (clarity, X_9:2_ = 1) and demonstrates protrusions of economic-ecological balance in the X_6_ (Policy Field) dimension (X_6:2_ = 1, X_6:4_ = 1), yet exhibits shallow depressions in X_5_ (Policy Targets; 0.40/1) and X_7_ (Policy Function; 0.33/1). While the policy meets the requirements of policy legitimacy theory through detailed goal-setting (X_9:2_ = 1), it lacks bottom-up participation mechanisms, specifically manifested by the exclusion of non-governmental stakeholders (X_5:5_ = 0). Although the policy reflects the Sustainable Development Goals (SDGs) by linking the environment and economy, it fails to operationalize SDG 17 (Partnerships) by omitting private sector participation (e.g., enterprise tax incentives). Empirically, the vague definition of “grassroots public sectors” (X_5:2_ = 1 without clarified responsibilities) has led to overlapping duties among village committees, significantly increasing administrative costs; meanwhile, the absence of “eco-tourism” clauses (as in P2) has limited economic spillover effects, with annual rural tourism revenue growth significantly reduced.

P7 (Yinchuan City, PMC = 5.73): incentivized but culturally blind. Its surface morphology (Figure 8) shows moderate protrusions in the X_4_ (Policy Type) dimension (mixed incentives, X_4:1_ = 1, X_4:2_ = 1) and significant elevation in X_7:5_ (agricultural development, 1), yet exhibits broad depressions in X_1_ (Policy Nature; 0.67/1) and X_7:4_ (cultural preservation, 0.). While the policy applies behavioral economics through “eco-points” rewards (X_4:2_ = 1), it overlooks cultural theory, as illustrated by toilet renovation designs that failed to incorporate local architectural traditions (X_7:4_ = 0). Although aligning with agricultural modernization goals (X_7:5_ = 1), it lacks cross-sectoral integration, failing to link waste management with rural cultural tourism. Empirically, the absence of feedback mechanisms (X_1:3_ = 0) led local villagers to reject new toilets and revert to traditional facilities due to cultural mismatch; while “organic fertilizer subsidies” (X_7:5_ = 1) enhanced agricultural productivity, the lack of ecological culture programs (e.g., folklore-themed waste education) constrained long-term behavioral change and hindered effective recycling of agricultural waste.

#### 4.4.3 Morphological and policy analysis of unsatisfactory level policy

The “Unsatisfactory” policy group (P4, P6) exhibits fundamental design flaws, with PMC indices below 5.0 (4.91 and 4.53, respectively), reflecting systemic gaps in policy integrity, stakeholder inclusivity, and contextual adaptability. Their PMC surfaces are characterized by severe, multi-dimensional depressions, with only minimal protrusions in policy type (X_4_) and limited policy fields (X_6_). The following strengthened analysis integrates theoretical frameworks and empirical evidence to address prior gaps. The detailed analysis is as follows:

P4 (Yuncheng City, PMC = 4.91): rigid institutionalism without adaptive capacity. Its surface morphology (Figure 5) is dominated by a single protrusion in X_4_ (Policy Type; mandatory tools, X_4:1_ = 1) and marginal elevations in X_6_ (Policy Field; ecology, X_6:4_ = 1), yet exhibits significant depressions across X_1_ (Policy Nature; 0.50/1), X_2_ (Policy Timeliness; 0.33/1), X_5_ (Policy Targets; 0.40/1), and X_7_ (Policy Function; 0.33/1). The policy relies entirely on command-and-control governance (X_4:1_ = 1), aligning with traditional institutional theory but violating adaptive governance principles due to the lack of feedback mechanisms (X_1:3_ = 0). It also fails to operationalize stakeholder theory, notably by excluding educational institutions (X_5:5_ = 0) and civil society, reflecting a “state-centric” model inconsistent with multi-stakeholder governance norms. Empirical evidence reveals specific shortcomings: (1) conceptual vagueness: The policy’s definition of “rural environmental sanitation” (X_1:5_ = 0.57) excludes agricultural non-point source pollution, leading grassroots actors to focus only on superficial waste cleanup while neglecting pesticide packaging recycling; this oversight has significantly increased farmland pollution incidents in targeted areas; (2) timeline ambiguity: The absence of mid/short-term plans (X_2:2_ = 0, X_2:3_ = 0) has delayed the construction of waste treatment facilities, preventing projects from achieving their intended outcomes. (3) Functional monotony: By focusing solely on “improving living environments” (X_7:1_ = 1) without cultural or economic linkages (X_7:4_ = 0, X_7:5_ = 0), the policy’s relevance has been weakened, with villagers perceiving initiatives as “government mandates” rather than community-driven goals.

P6 (Turpan City, PMC = 4.53): structural collapse due to contextual disconnect. Its surface morphology (Figure 7) exhibits a “pot-bottom” collapse across nearly all dimensions, with minimal protrusions only in X_4_ (Policy Type; X_4:1_ = 1) and X_6_ (Policy Field; technology, X_6:5_ = 1), yet critical depressions in X_1_ (0.33/1), X_5_ (0.60/1), and X_7_ (0.17/1). By replicating urban-centric models (e.g., centralized waste stations) in mountainous, scattered villages, the policy violates place-based governance theory and ignores geographic and socioeconomic realities. It also fails to integrate cultural theory, as demonstrated by toilet renovation designs that disregard local ethnic customs (X_7:4_ = 0), contradicting the importance of cultural legitimacy in policy acceptance. Empirical evidence reveals specific deficiencies: (1) feedback mechanism Failure: The complete absence of feedback loops (X_1:3_ = 0) rendered policies unable to adapt to village-specific challenges. For example, high transportation costs for centralized waste stations in mountainous areas led to facility idling or underutilization. (2) Stakeholder exclusion: Limiting targets to grassroots organizations (X_5:3_ = 1) while excluding educational institutions (X_5:5_ = 0) and private enterprises resulted in extremely low public participation, preventing effective coordination among various stakeholders. (3) Functional myopia: The policy’s sole focus on infrastructure (X_7:1_ = 1) neglects eco-economic synergies (e.g., waste-to-energy projects, X_7:5_ = 0), missing opportunities to integrate with rural tourism development.

### 4.5 Contrastive analysis

Among the seven policy samples, P2 distinguishes itself with a PMC index of 7.6—the only policy rated “excellent.” Its superiority lies in a holistic integration of policy design, stakeholder engagement, and contextual adaptation, as evidenced by its performance across all nine primary variables (Table 6). Unlike other policies, P2 achieves full or near-full scores in X_3_ (Policy Perspective), X_4_ (Policy Type), X_7_ (Policy Function), X_8_ (Supporting Measures), and X_9_ (Policy Quality), reflecting a rare balance of macro-strategic alignment and micro-implementation feasibility.

#### 4.5.1 Structural foundations of P2’s success

The remarkable effectiveness of the P2 policy stems from its meticulous design and implementation across multiple interlinked and mutually reinforcing dimensions (see Table 7).

**Table 7:** The comparison between the “excellent” policy and other policies.

Factor	Excellent level policy(P2)	Average of other policies
Feedback mechanisms	Explicit three-tier system	None or informal
Stakeholder breadth	4 sectors (government, educational institutions, enterprise, community)	2 sectors (government, community)
Timeline specificity	20 + measurable milestones	1–2 vague goals
Cultural adaptation	Local design elements integrated	Generic urban design
Economic linkages	Waste-to-energy, tourism	Isolated environmental goals

One of its core strengths lies in the establishment of a dynamic feedback and adaptive mechanism. P2 stands as the sole policy that explicitly incorporates a three-tier feedback system. At the village level, quarterly surveys conducted by community committees are used to promptly gauge villagers’ satisfaction with waste collection frequencies and the accessibility of facilities. At the township level, monthly meetings are held to adjust policies in response to geographical challenges flexibly; for example, GPS-tracked garbage trucks are utilized to optimize transportation routes in mountain villages. At the city level, annual reviews are carried out, comprehensively considering environmental indicators (such as the reduction in river pollution levels) and economic indicators (such as the growth in rural tourism revenue associated with sanitation improvements). Thanks to this mechanism, P2 successfully avoids the “one-size-fits-all” drawbacks seen in P6 and P4, where rigid urban models led to significant facility idling. In contrast, the adaptive design of P2 reduces operational costs and substantially increases service coverage in villages.

On another front, P2 pioneers a multi-stakeholder collaborative governance ecosystem involving the government, educational institutions, enterprises, and the community. As educational hubs, educational institutions mandate “environmental stewardship” programs in all K-12 institutions. These programs, which include waste-sorting competitions and annual village clean-up days, effectively enhance students’ environmental awareness and influence household environmental behaviors. Enterprises must allocate a certain proportion of their yearly profits to the construction of rural sanitation infrastructure, and compliant enterprises can enjoy tax incentives, which attract a large amount of private investment. At the community level, the “eco-points” system rewards households for proper waste segregation, with points redeemable for daily necessities or agricultural supplies, significantly boosting residents’ participation.

Simultaneously, P2 translates its long term goal of “achieving sustainable sanitation by 2025” into a phased framework. The 2021–2022 period is the pilot phase, during which solar-powered waste stations are installed in selected model villages, and professional village sanitation supervisors are trained. From 2023 to 2024, the expansion phase is implemented, with centralized sewage treatment systems rolled out in most townships and connected to real-time monitoring systems. By the consolidation phase 2025, the goal is to achieve comprehensive waste sorting and a high sewage treatment rate, with village self-governance committees overseeing operations. This rigorous phased planning stands in sharp contrast to the situation in P1, where the lack of clear goals led to significant delays in project construction and ensured the smooth achievement of key performance indicators.

Moreover, successfully implementing the P2 policy in Jiujiang demonstrates the importance of contextual adaptation. Jiujiang reconciles the universality of policies with regional specificity. Regarding geographical diversity, decentralized biogas systems are adopted in mountainous areas to handle organic waste, significantly reducing transportation costs, while automated sorting plants in plain areas efficiently process mixed waste. Economically, the policy integrates sanitation improvements with rural tourism development by creating “Eco-Villages,” which have increased tourism revenue. Culturally, local architectural elements are incorporated into the public facilities of traditional villages, earning high levels of community acceptance. All these aspects further attest to the flexibility and effectiveness of the P2 policy.

#### 4.5.2 Transferable lessons for policy improvement

To systematically enhance policy effectiveness and facilitate cross-regional experience transfer, the successful practice of Policy P2 reveals three universally applicable improvement pathways that form a replicable policy upgrading framework through the organic integration of institutional design, stakeholder coordination, and implementation optimization:

First, it is essential to establish an institutionalized feedback and learning system to shift policies from experience-based trial-and-error to scientific iteration. This involves not only developing standardized full-cycle evaluation mechanisms covering the entire process of “policy formulation-piloting-promotion-evaluation” and integrating quantitative indicators with qualitative feedback but also establishing cross-level “policy innovation laboratories” to build regional learning networks and developing policy simulation systems to enable intelligent adjustments through digital technologies. For example, in agricultural non-point source pollution control, quarterly evaluations combining satellite remote sensing monitoring with farmer interviews could be introduced, while in crop straw burning governance, a “dynamic subsidy + technological substitution” package could be refined for cross-regional adaptation.

Second, innovating multi-stakeholder collaborative governance models is critical to transition from government-dominated implementation to pluralistic participation. This requires not only integrating environmental governance into the national education system and strengthening the policy transmission function of educational institutions through tiered curricula but also establishing a positive feedback mechanism of “environmental contribution-economic return” to incentivize corporate participation and promoting the “ecological points bank” model to enhance community self-governance via digital platforms for cross-village point circulation and value-added. Examples include K-12 “small hands pulling big hands” waste-sorting competitions, corporate carbon credit trading, and linking points to rural tourism reception qualifications.

Third, optimizing the temporal–spatial policy implementation framework is necessary to move from vague and simplistic approaches to precise implementation. This involves adopting a three-tiered framework of “long-term vision-medium-term planning-short-term actions” in the temporal dimension, constructing a “regional characteristics-policy tools” adaptation matrix to address geographical, economic, and cultural differences in the spatial dimension, and establishing standardized policy terminology dictionaries to avoid semantic ambiguity. For instance, different waste management models could be designed for mountainous and plain areas, differentiated incentive mechanisms implemented in poor and developed regions, and technical standards clarified for “rural domestic waste management” through a four-tiered system.

In summary, learning from Policy P2’s success requires adopting its specific measures and internalizing its governance philosophy. P2’s experience essentially reveals three core principles of modern policy governance: policies must possess dynamic adaptability to “perceive the environment-self-repair-evolve and upgrade” like living systems; governments must shift from “implementers” to “ecosystem builders” to activate synergistic stakeholder engagement; and policies must be “rooted” in specific geographic and sociocultural contexts while preserving room for local innovation within universal frameworks. These principles extend beyond environmental governance to provide methodological references for optimizing policy systems in rural revitalization, grassroots governance, and other domains.

## 5 Discussion and conclusion

### 5.1 Key findings and implications

This study adopts the PMC index model combined with text-mining technology to quantitatively evaluate the policy texts of rural environmental sanitation governance promulgated by 7 cities in China. It focuses on analyzing each policy’s consistency, advantages, and disadvantages, and the main findings are as follows.

First, the content design of these 7 policies is generally acceptable. The average PMC index of the 7 policies is 6.03, with relatively good consistency. The development of each indicator within the policy is relatively balanced, and the overall structure is reasonable. Among the 7 policy samples, 1 policy is rated as excellent, 4 as good, and 2 as unsatisfactory.

Second, the analysis identifies persistent weaknesses in four primary variables: Policy Nature (X_1_), Timeliness (X_2_), Targets (X_5_), and Functions (X_7_). For instance, most policies lack precise feedback mechanisms, conceptual definitions, and phased timelines (short/medium-term plans), which hinder adaptive governance and practical implementation. Additionally, stakeholder targets often overlook educational institutions, limiting public participation and environmental awareness campaigns. Policy functions predominantly focus on infrastructure improvement (e.g., waste treatment) while neglecting cultural and agricultural development linkages, such as rural cultural enrichment or eco-agriculture promotion. These gaps suggest a need for policy frameworks to adopt a more holistic approach, integrating dynamic adjustment mechanisms and multi-stakeholder collaboration.

### 5.2 Theoretical and practical contributions

This study advances the field of policy evaluation by introducing the PMC Index Model into rural environmental governance, addressing the scarcity of micro-level quantitative analyses of policy texts, and enriching the literature on policy assessment. Unlike traditional qualitative approaches or post-implementation outcome studies, this methodology systematically dissects policy components (e.g., timeliness, stakeholder inclusivity) through data-driven text mining, offering a replicable framework for evidence-based policy design. The research enables cross-regional comparisons by visualizing policy strengths and weaknesses via three-dimensional PMC surfaces. It identifies structural flaws contributing to governance inefficiencies, challenging the assumption that implementation failures account for poor governance outcomes.

For policymakers, the findings highlight the urgency of enhancing policy integrity through targeted improvements. Regarding the dimension of policy nature (X_1_), feedback mechanisms and clear conceptual descriptions should be incorporated to improve policy flexibility and public understanding. Regarding timeliness (X_2_), short- and medium-term milestones should be set in addition to long-term goals to facilitate phased implementation and accountability. As for the dimension of stakeholder targets (X_5_), the scope of stakeholder engagement should be expanded to include educational institutions, non-governmental organizations, and community groups, thereby promoting bottom-up participation. In the dimension of policy functions (X_7_), environmental education, cultural preservation, and agricultural modernization objectives should be integrated to create synergistic governance effects.

### 5.3 Limitations and future directions

This study has certain limitations. First, the limited sample size of seven policies introduces significant constraints on generalizability. While the study explicitly focuses on cities promulgated dedicated rural environmental sanitation management measures, excluding provincial-level policies and pilot programs may skew results toward local-level idiosyncrasies rather than national trends. For instance, the absence of policy documents from economically underdeveloped regions (e.g., Tibet, Qinghai) limits the ability to assess how resource scarcity influences policy design. This gap may lead to overgeneralization of findings, particularly regarding the correlation between per capita income and policy quality observed in the sample.

Second, while this study incorporates text mining and literature review in variable design, the evaluation dimensions and variable selection still exhibit subjectivity. Future research could employ grounded theory to systematically derive deeper dimensions and variables, thus establishing a more scientific evaluation framework.

Finally, methodologically, while the PMC index model analyzes policy content consistency and advantages/disadvantages, the results focus primarily on document-level content quality and lack a close connection to policy implementation outcomes. This limitation is inherent in the PMC index model. Future research may integrate complementary methodologies to address this gap.

## 6 Conclusion

This research provides a rigorous framework for evaluating rural environmental policies, demonstrating the PMC Index Model’s utility in identifying structural flaws and best practices. By prioritizing feedback-driven adaptability, inclusive stakeholder engagement, and phased implementation, policymakers can bridge the gap between policy objectives and rural development needs, fostering sustainable and equitable environmental governance. The study’s methodology and recommendations offer a replicable template for evidence-based policy design in developing countries facing similar rural environmental challenges.

## Data Availability

The original contributions presented in the study are included in the article/supplementary material, further inquiries can be directed to the corresponding author.
